# Supplementary material for: Cloud BioLinux: pre-configured and on-demand bioinformatics computing for the genomics community
Source: BMC Bioinformatics. 2012 Mar 19;13:42. doi: 10.1186/1471-2105-13-42 (PMC3372431; doi:10.1186/1471-2105-13-42)
Supplement: Additional file 1 — Supplementary 1 Cloud BioLinux software documentation in the form of a mini, self-contained website. Users need to download and uncompress the .zip file, and open through a web browser the "index.html" file available on the main directory. (ZIP 1823 kb). [file 1471-2105-13-42-S1.ZIP › Cloud-BioLinux-Package-Documentation/docs/t-coffee.html]

Bio-Linux Software Documentation Pages

Back to search form

## t-coffee

|  |  |
| --- | --- |
| Name | t-coffee |
| Description | **T-Coffee** is a multiple sequence alignment package. Given a set of sequences (Proteins or DNA), T-Coffee generates a multiple sequence alignment.  **References:**  T-Coffee: A novel method for multiple sequence alignments. C.Notredame, D. Higgins, J. Heringa,Journal of Molecular Biology,Vol 302, pp205-217,2000 |
| Homepage | http://igs-server.cnrs-mrs.fr/~cnotred/Projects\_home\_page/t\_coffee\_home\_page.html |
| Remote Documentation | http://igs-server.cnrs-mrs.fr/~cnotred/Documentation/t\_coffee/README |
